# A fungus among us: The emerging opportunistic pathogen *Candida tropicalis* and PKA signaling

**DOI:** 10.1080/21505594.2018.1438026

**Published:** 2018-03-19

**Authors:** Anuj Kumar

**Affiliations:** Department of Molecular, Cellular, and Developmental Biology and Program in Cellular and Molecular Biology; University of Michigan; Ann Arbor, MI, USA

**Keywords:** *Candida tropicalis*, cAMP-dependent protein kinase A, *Candida albicans*, hyphal development, filamentous growth, pathogenesis, Ras, virulence

Opportunistic infections by *Candida* species remain a significant and costly health concern across a broad swath of the population. Preterm infants, with their immature immune systems, are at a high risk for potentially fatal infections from several species of *Candida* [1]. Adults with weakened immune systems, both from diseases such as HIV and from chemotherapy treatments, are susceptible to painful candidial infections, including oral thrush [2]. Recent data indicate that *Candida* infections account for 80% of all systemic fungal infections worldwide with *C. albicans* identified as the most commonly isolated *Candida* species [3]. In this respect, however, the fungus *Candida tropicalis* has also emerged as an important opportunistic fungal pathogen. On a worldwide scale, incidents of *C. tropicalis* infections have risen over the past two decades, and recent reports have identified strains resistant to commonly administered azole drug treatments, such as fluconazole [4-6]. *C. tropicalis* has been identified as the most frequently observed clinically isolated yeast in Asia and is among the three most prevalent yeasts found in superficial and systemic infections in Latin America [7,8]. Considering its understudied biology and associated virulence, *C. tropicalis* is a key subject for further research.

*C. tropicalis* was first isolated more than a century ago from a patient in the tropics exhibiting symptoms of a bronchial infection [9]. *C. tropicalis* is a commensal organism but, as its name suggests, is also found in the environment distributed widely in tropical and sub-tropical marine settings in seawater and on beaches [10]. Phylogenetic analysis suggests that *C. tropicalis* is fairly closely related to *C. albicans*. Perhaps consequently, *C. tropicalis* exhibits several phenotypic traits associated with *C. albicans*. *C. tropicalis* can produce true hyphae like *C. albicans* and *C. dubliniensis* and efficiently forms biofilms [11]. *C. tropicalis* is adherent to epithelial and endothelial cells [12]. The ability to switch between white and opaque cell types is also evident in *C. tropicalis*, with the morphological plasticity of this fungus relevant for its ability to undergo sexual mating and its associated virulence [13].

Numerous virulence factors have been identified in *Candida* species, and the signaling pathways enabling morphogenetic switching from yeast-like to filamentous growth forms have been well studied in this regard. The ability to transition between morphological states, in species such as *C. albicans*, is thought to be critically important in establishing several processes associated with virulence, including epithelial cell invasion, endothelial rupture, evasion from phagocytic cells, and biofilm formation [14]. As a result, substantial attention has been focused upon the signaling pathways that regulate these morphological transitions, with much of the research occurring in *Candida albicans* and in a filamentous strain of the baker's yeast *Saccharomyces cerevisiae*.

The rat sarcoma (Ras)/protein kinase A (PKA) pathway is an established regulator of fungal filamentous-form growth. In *S. cerevisiae*, landmark studies from the 1980's established Ras2p as an activator of adenylate cyclase, which produces cyclic adenosine monophosphate (cAMP) [15]. cAMP binds to the regulatory subunit of PKA, releasing one of three catalytic isoforms of the kinase, Tpk1p, Tpk2p, or Tpk3. The *S. cerevisiae* forms of PKA differ in their filamentous growth phenotypes: deletion of *TPK1* does not impact pseudohyphal growth; deletion of *TPK2* results in decreased filament formation; and deletion of *TPK3* results in exaggerated pseudohyphal growth [16]. Tpk2p phosphorylates the transcription factor Flo8p, which is required for wild-type pseudohyphal growth [17]. Much of the data discovered from studies of *S. cerevisiae* is also relevant in understanding PKA biology in *C. albicans*; however, some distinctions do exist. *C. albicans* encodes two PKA catalytic subunit isoforms, Tpk1p and Tpk2p. Although functions for these PKA subunits have not come into precise focus yet within the *C. albicans* research community [18], Tpk1p and Tpk2p are generally recognized to produce partially overlapping loss-of-function phenotypes, with *TPK1* required for filamentation on solid medium but dispensable for filamentation in liquid, and *tpk2* mutants exhibiting an opposite phenotype [19]. Further, in *C. albicans*, *TPK2*, but not *TPK1*, is required for virulence in murine models of candidiasis [20]. The *C. albicans* gene *CYR1*, which encodes adenyl cyclase, is non-essential, while its *S. cerevisiae* ortholog is required for cell viability under standard laboratory growth conditions [21,22]. Accordingly, recent studies indicate that PKA is non-essential in *C. albicans*, as a mutant strain deleted of both *TPK1* and *TPK2* is viable [18]. Collectively, though, the PKA pathway in both fungi is required for wild-type regulation of filamentation and may constitute an antifungal drug target in *C. albicans* [23].

The studies and data above underscore the importance in studying PKA signaling in additional medically relevant fungal systems. In this issue, Lin and colleagues investigate the role of PKA with respect to filamentous development and virulence in *C. tropicalis* [24]. Like *C. albicans*, *C. tropicalis* encodes two catalytic subunit isoforms of PKA, Tpk1p and Tpk2p. In a previous study, single deletion mutants of either gene in *C. tropicalis* had indicated redundant functions in filamentation, with both mutants exhibiting wild-type glucose-induced filamentous development. The work by Lin *et al.* presents a phenotypic analysis of both single and double deletion mutants in *C. tropicalis* for phenotypes related to hyphal development, stress tolerance, biofilm formation, and virulence. From these analyses, a mutant deleted of both *TPK1* and *TPK2* was viable but exhibited severely impaired growth relative to wild type at 30°C and 37°C in standard media, with growth abolished at more extreme temperatures of 25°C and 42°C. These growth defects were partially rescued by complementation with either *TPK1* or *TPK2*. As in *C. albicans*, Tpk2p contributes more strongly to PKA activity than Tpk1p in *C. tropicalis*. Using the PepTag assay, *TPK2* deletion resulted in decreased substrate phosphorylation, while the homozygous *tpk1* mutant exhibited PKA substrate phosphorylation comparable to that observed in wild type.

Lin and colleagues further distinguished functions of *TPK1* and *TPK2* in *C. tropicalis* through phenotypic assays for stress responses, cell morphogenesis, and adhesion. In contrast to the *tpk2* mutant, a homozygous diploid strain deleted for *TPK1* exhibited hypersensitivity to several antifungal drugs, cell wall perturbing agents, and to treatment with 3-amino-1,2,4-triazole (3-AT). The *tpk2* deletion mutant exhibited impaired growth relative to wild type upon exposure to 0.85 M magnesium chloride. Homozygous mutants deleted of *TPK2* exhibited diminished hyphal development relative to wild-type and homozygous *tpk1* deletion strains on media supplemented with either N-acetylglucosamine (GlcNac) or 3-AT, and a similar phenotype was evident on nitrogen-limiting SLAD media. Interestingly, on SPIDER medium with mannitol as a carbon source, *tpk2* deletion mutants were hyperfilamentous relative to *tpk1*mutants and wild type. Further assays by Lin and colleagues identified *tpk2* deletion mutants as exhibiting defects in sedimentation relative to wild type and *tpk1* mutants, with the phenotype being lost in a homozygous *tpk1 tp2* double deletion mutant. Neither *TPK1* nor *TPK2* were required for adhesion to plastic; however, *TPK2*, but not *TPK1*, was required for biofilm formation on polystyrene.

Considering these phenotypes relevant to virulence, Lin and colleagues investigated the role of PKA in *C. tropicalis* pathogenicity using a murine model of systemic infection. By these analyses, mutants doubly deleted of *TPK1* and *TPK2* exhibited significantly reduced virulence, decreased fungal burden in the brain and kidneys, and decreased fungal cells and necrotic tissue in histopathological samples. Single deletion mutants exhibited phenotypes resembling wild type, indicating that the catalytic subunits function redundantly with respect to *C. tropicalis* virulence.

Collectively, the study by Lin *et al.* clarifies the role of PKA and its constituent catalytic subunit isoforms in regulating cell growth, stress response, and virulence. Additional work remains in delineating the mechanisms underlying these phenotypes, which may be complex in light of the many regulatory interconnections likely between PKA and other signaling pathways. The research presented in the paper by Lin and colleagues, however, underscores the importance of such studies, as the PKA pathway in *C. tropicalis* is undoubtedly a critical signaling component in the biology and virulence of this emerging opportunistic human fungal pathogen.
